# Labour and Health.—II. Wanted, More Air

**Published:** 1894-09-29

**Authors:** 


### Modern Sociology.

### II.—WANTED, MORE AIR.

One of the most baffling elements in a complex civilisation is the terrible difficulty of doing good without creating some new evil. Almost every great reform has brought in its train a host of minor evils, and some have been met and vanquished in the height of their triumph by a Frankenstein monster, for whom alone they are responsible. It has taken a good many years to test the new methods of modern philanthropy in the direction of housing the poor, and only quite recently has any misgiving about these rashly-named "model dwellings" been forced upon the public. Mr. Robert Williams,* in his recent pamphlet on the subject, goes far to turn the misgiving into unhappy certainty of failure. Unqualified failure no one could for a moment ascribe to the men and women who have laboured in this great cause. Bearing in mind the immaculate water-supply of the most jerry-built model of to-day, its drainage better by far on the whole than that of Belgravia, and the moral gain alone implied in the estimate of 3·5 to a room which Mr. Williams supplies of the population in the most crowded and reprehensible buildings cited, the very idea of failure seems at first sight inexplicable. Pure water, good drainage, new ideals of decency, and a higher standard of comfort and convenience are no small boons. Unfortunately, in the desire to confer them on as large a number as space would permit, the promoters of model dwellings have reckoned without one simple factor of nature, which is now, in nature's usual way, exacting a terrible revenge. They forgot that their lodgers must breathe. It is not that ample provision was not made for ventilation. There are plenty of windows in the models and good care is taken that they shall open top and bottom. The fault is in the air itself. And strange as it may seem, the discovery has been made too late that there is not enough of it to go round, not enough, that is to say, over one acre of ground to supply the lungs of 3,600 persons. What this population means (and it is exceeded in some of the new blocks of workmen's dwellings) may be realised by Mr. Williams' calculation that it allows a space of about a yard and a quarter for each person, and the corresponding calculation of Dr. Richardson that in a city built under perfect sanitary conditions the number of persons to an acre must not exceed twenty-five.

The extent to which the atmosphere is vitiated in and round these monstrous erections has been very accurately tested by Mr. W. C. Young, F.I.C. The amount of carbonic acid present per thousand by volume was found in 42, Wentworth Builings to be 0·967, and the mean of four specimens of inside air was 0·853. The results of the examination of the outer air were even more startling. Whereas the worst specimens of outer air taken thirty years ago by Dr. Angus Smith at Smithfield showed a pollution of 0·428 of carbonic acid per 1,000, the average taken this year at Whitechapel showed 0·528, or an increase of carbonic acid of 0·140 per 1,000. Ventilation, then, in these regions means the process of diluting the "0·9 per 1,000 of carbonic acid by the 0·5 per 1,000, so as to make it, say, 0·7 per 1,000," and this is the result of thirty years of incessant effort at sanitary improvement.

Bad air is by no means so speedy in making its effects felt as bad water. It takes a long time to kill, but kill it does in the end. The death-rate is an infallible danger signal, and its warnings cannot be better expressed than by reproducing the following table, compiled from reports of the Registrar and Medical Officer.

| | Population. | Density per acre. | Death-rate per 1,000. |
|---|---|---|---|
| London and suburbs ... ... | 4,263,294 ... | 57· 7 ... | 23·2 |
| Whitechapel ... ... ... | 74,714 ... | 196· 0 ... | 25·0 |
| Borough Road, sub-district... | 16,224 ... | 259· 7 ... | 32·0 |
| St. Giles', South, sub-district | 13,697 ... | 214·01 ... | 35·6 |
| | | | Average for 10 years. |
| Four model blocks in Whitechapel ... ... ... | 2,380 .. | 3,000 ... | 41·4 |

Further evidence as to the evils of over-crowding seems superfluous after this. But the recent rapid spread of scarlet fever and small-pox, and the appalling prevalence of phthisical complaints, form no small part of Mr. Williams' indictment against the model dwelling. That the inhabitants undergo a steady deterioration of all their vital powers is unhappily only too evident, and the direct loss to the nation involved in this deterioration outweighs any monied interest which may be at stake. Everyone has tested the effect on the health, energy, and moral forces of fresh air, and has experienced the strange depression and sinking of the heart which a polluted atmosphere, endured for a few hours only, will produce. It is inconceivable tnat under these conditions the best work should be done, that men and women can maintain a decent standard of living, or that a healthy race can be reared for the credit of the nation.

* "More Light and Air for Londoners." By Robert Williams A.R.I.B.A. (William Reeves.)

